# Gastrointestinal Complications of Laparoscopic/Robot-Assisted Urologic Surgery and a Review of the Literature

**DOI:** 10.14740/jocmr2090w

**Published:** 2015-02-09

**Authors:** Mert Ali Karadag, Kursat Cecen, Aslan Demir, Murat Bagcioglu, Ramazan Kocaaslan, Teoman Cem Kadioglu

**Affiliations:** a Department of Urology, Faculty of Medicine, Kafkas University, Kars, Turkey; b Department of Urology, Istanbul University, Medical Faculty of Istanbul, Istanbul, Turkey

**Keywords:** Bowel, Complications, Gastrointestinal, Laparoscopic surgery, Robotic surgery, Trocar, Urology

## Abstract

Gastrointestinal injuries that occur during or after laparoscopic and robot-assisted surgery are serious side effects that affect patient outcome. In this review, we attempt to highlight the identification, incidence and management of gastrointestinal and visceral complications of laparoscopic and robot-assisted surgery. A search of Medline and PubMed databases was performed using the following terms: gastrointestinal complications of laparoscopy, laparoscopic, kidney and robotic surgery. A total of 1,072 papers related to the subject were analyzed. Forty-six of these papers were included in the present review. These papers reported high numbers of participants and had a high level of evidence. Gastrointestinal complications during laparoscopic and robot-assisted surgery are rare, but similar, and can occur at any time between access and closure. Despite their infrequency, these complications can result in mortality. The early recognition and management of gastrointestinal complications is very important. Unrecognized or delayed identification of gastrointestinal complications may cause sepsis and death.

## Introduction

Laparoscopic approaches for urologic pathologies have become popular and are common all over the world. Laparoscopy has numerous advantages such as a shorter hospital stay and convalescence, lower patient morbidity and decreased blood loss when compared with conventional surgery.

The reports from large multi-institutional studies focusing on urologic laparoscopy revealed an overall complication rate from 4.4% to 16% and a mortality rate up to 0.9% [1-5]. Gastrointestinal injuries during or after laparoscopic and robot-assisted surgery are serious side effects that may occur in addition to urologic and vascular complications. The reported incidence of bowel injury is approximately 1.3 per 1,000 cases [6].

In this review, we highlight the identification, incidence and management of gastrointestinal and visceral complications of laparoscopic and robot-assisted surgery.

## Construction and Content

A search of Medline and PubMed databases was performed using the following terms: gastrointestinal complications of laparoscopy, laparoscopic, kidney and robotic surgery. A total of 1,072 papers related to the subject were analyzed. Only 46 of these papers were included in the present review. These papers reported high numbers of participants and had a high level of evidence. The retrieval time ended in August 2014.

## Utility and Discussion

### Gastrointestinal effects of pneumoperitoneum

Halevy et al compared laparoscopic and open surgery in terms of gastrointestinal motility. They concluded that laparoscopic surgery disrupted motility less than open surgery [7]. The mechanism behind the decreased occurrence of ileus after laparoscopic surgery remains unclear. Gastroesophageal reflux is another issue following abdominal laparoscopy. Despite the increased intra-abdominal pressure due to insufflation, the incidence of gastroesophageal reflux and regurgitation in patients undergoing laparoscopic surgery is not increased [8]. The Trendelenburg position, especially during radical prostatectomy, morbid obesity, and high intra-abdominal pressure during laparoscopy can facilitate gastroesophageal reflux, regurgitation and aspiration of the gastrointestinal contents. Intravenous metoclopramide (10 mg) can be administered preoperatively to high risk patients to avoid reflux [9]. Alpha blockers and a cuffed endotracheal tube can prevent patients from the aspiration of gastric contents.

### Access-related gastrointestinal injuries

A bowel injury can occur at anytime during laparoscopy from access to closure. A recent meta-analysis showed that the incidence of gastrointestinal tract injury during laparoscopy was 0.13% [10]. The small intestine was the most commonly injured bowel portion, with an injury rate of 41.8%; the predominant reason for this injury was due to gaining access to the abdomen using either a Veress needle or trocar. The Food and Drug Administration (FDA) reported 639 trocar-related complications from 1993 to 1996. Bowel injuries accounted for 134 (21%) of these cases [11], and six (4.5%) injuries were unrecognized and resulted in patient death.

Correct Veress needle placement should be evaluated before insufflation of the abdomen. The stomach or bowel may be entered during access with a trocar or Veress needle. Transgression into a hollow viscus is immediately apparent because of the typical appearance of the gastrointestinal contents [12]. The bowel may be insufflated with a Veress needle, and this may cause asymmetrical abdominal distension. The passage of flatus during insufflation must be considered problematic because only a small amount of CO_2_ (< 2 L) insufflation is required to reach a high intra-abdominal pressure. The insufflation should be stopped if any of these signs occur, and access with a Veress needle or an open (Hasson) technique should be performed.

If a bowel injury is recognized at the time of surgery, the management required depends on the severity of injury. Conservative treatment may be an option if the Veress needle perforates an intra-abdominal organ and no enteric content or a hollow viscus is observed. On the other hand, laparoscopic intracorporeal suturing techniques or open surgery based on the experience of the operating surgeon can be used for repairing the injury. A general surgery consultation may be required for determining the severity of the injury and management strategies.

The primary trocar, which is not inserted under direct visual guidance, is responsible for most trocar-induced bowel injuries. When the bowel is injured by a trocar, the surgeon should not remove the trocar immediately; it can be used to minimize the leakage of intestinal contents into the peritoneum [12]. Thus the primary trocar location should always be checked to detect potential intra-abdominal or bowel injuries.

Trocar insertion during upper abdominal accesses may also result in gastric perforation. Fasting for 8 h prior to surgery and the insertion of an oro- or nasogastric tube can minimize the risk of gastric injury [12].

The placement of ports in transperitoneal laparoscopic renal surgeries is as follows: A 10/12 mm port is inserted on the mid-clavicular line just at or above the upper border of the umbilicus. A 10/12 mm working port is placed a finger below the costal margin on the anterior axillary line. A 10/12 mm or 5 mm second working port can be placed on the anterior axillary line just above the superior iliac crest and an additional port for a retracting instrument may be inserted on the mid-axillary line mid-way between the superior iliac crest and the costal margin. This is the most suitable port placement map for patients with normal and high body mass index (BMI). Herein, some specific recommendations are given with regards to port placements like if the patient to be operated on is extremely thin; all of the ports should be removed medially to avoid any complications during trocar placement. By positioning the patient so that the spleen and liver are lateral, injury of these organs with the passage of the primary trocar is unusual [13]. Trocar injuries of the urinary tract are an uncommon complication, but were reported by Ostrzenski and Ostrzenska in 1998. The bladder was injured and managed by laparoscopic suturing techniques and a tissue stapler. The authors advise preoperative insertion of a urethral catheter to drain the bladder prior to all major laparoscopic cases to prevent this problem [14].

Abdominal access in patients with prior abdominal surgery or extreme BMI (high or low) may be challenging. Some authors advised the use of non-bladed (dilating) trocars to minimize the risk of bowel and vascular injury in these patients [15, 16]. In a recent study, transperitoneal robotic partial nephrectomy (RPN) outcomes were evaluated in a total of 95 patients that had localized renal cell carcinoma, of which 41 (43%) had a history of previous abdominal surgery and six had upper midline or ipsilateral upper quadrant scars. They reported an enterotomy in a patient during the lysis of adhesions that was repaired with a robot without sequelae and a mesenteric hematoma during Veress needle placement [17].

Another access method in laparoscopy is known as the open or Hasson technique [18]. It is a safe method that can be used for patients with prior abdominal surgery, very low BMI, and children as well as if there is difficulty in establishing a pneumoperitoneum with a Veress needle. Like access with a Veress needle, vascular and bowel injuries have been reported with the open method [18-20].

Despite the technical differences between the two entry techniques, three prospective, randomized trials revealed that trocar insertion by a Veress needle is not more dangerous than direct visual trocar insertion [21-23]. Regardless of the technique chosen, the surgeon’s experience and skill is a key point. The following special points during surgery should be noted: irrigation through the needle should be easy, a positive drop test should be established (fluid dropped into the tip of the needle passes into the peritoneal cavity easily), no blood or fecal matter should be aspirated through the needle and initial pressure after entry should always be low. It is mandatory that the surgeons dealing with laparoscopy must know these two entry techniques very well.

### Non-access-related injuries of the bowel

A retrospective review on bowel injuries occurring during urologic laparoscopic surgery (915 cases from two institutions) reported an overall incidence of 0.8% non-access-related bowel injuries [6]. Serosal injury of the intestine or stomach occurred in six cases (0.6%), and bowel perforation was observed in two patients (0.2%). Intraoperatively recognized bowel injuries occurred during six surgeries: one during nephrectomy, three during pelvic lymph node dissection and two during pyeloplasty. Non-recognized bowel perforation occurred in two patients, and two additional patients who had non-recognized bowel perforations were referred from other institutions. As a result, three cases had a rapid progression to sepsis and two died. The patients with non-recognized bowel perforations did not have traditional peritoneal signs. The initial presentation was increased pain at a single trocar site without any discharge or erythema, followed by mild/severe leukopenia. Furthermore, one patient associated fever higher than 38 °C. Abdominal distension and diarrhea were also observed. The painful trocar site was closest to the injured bowel segment. This group also performed a literature search regarding bowel injuries during laparoscopic surgical or gynecological operations. They found a total of 12 access or non-access-related bowel injuries with an incidence of 0.13%. Most of the injuries (69%) were not recognized during the laparoscopic procedure.

Bowel injuries can occur during single site laparoscopic procedures. In a recent German publication, the clinical outcomes of laparo-endoscopic single site radical nephrectomy (LESS-RN) were reported [24]. The data included 42 patients who underwent LESS-RN between 2008 and 2011. The recorded adverse event included one bowel injury, which was repaired intraoperatively without requiring stoma.

Another speculative issue is that the low metabolic and immune response found after laparoscopic surgery allows for a quick progression into sepsis, when compared with open surgery [25, 26]. Other reasons may include the decreased stimulation of acute phase reactants, the small skin incisions of laparoscopy and a diminished metabolic and cytokine response after laparoscopy [12].

If the colonic injury is recognized intraoperatively, the bowel should be immediately repaired. An experienced surgeon can safely perform laparoscopic repair of the large bowel with intracorporeal suturing without a colostomy. Besides this, a diverting colostomy should be considered for preoperatively, unprepared patients with colonic injuries that require segmental bowel resection. It should be kept in mind that open laparotomy is required for nearly all unrecognized intraoperative injuries that present during the postoperative period [12].

If a bowel injury is suspected during the postoperative period, abdominal computerized tomography (CT) with a contrast agent should be performed immediately. It can help identify postoperative bleeding, bowel perforation, urinoma or urinary tract obstructions. The local thickness of the bowel and contrast agent in the peritoneal cavity should be absolute criteria for immediate exploration [12].

### Bowel injuries caused by electrocautery

Electrocautery is the second most common cause of intraoperative bowel injury [10]. These injuries cause serious complications, and most of the thermal damages to the bowel are not recognized during laparoscopy. The most dangerous type of injury is bowel-related and results from monopolar electrosurgical current. This injury can result from unwanted energy transfer into the operational field or an unrecognized current outside of the laparoscopic surgeon’s view. Unintended activation may cause direct application of the energy into a non-target tissue. Insulation breaks along the instruments are another cause of electrosurgical injury [12].

Sepsis and acute abdominal pain are typically observed 1 - 2 days after surgery. Nausea, trocar site pain, fever, leucopenia and chills are also signs. The clinical scenario can be challenging, and patients may quickly deteriorate. A CT scan can reveal extraluminal feces and/or free air in the abdomen. Additional imaging methods such as gastrograffin enema can also be used for the detection of an injured site [27].

The prevention of electrosurgical injuries is very important. Avoiding the application of monopolar energy sources and actively observing the location of instruments with the potential to injure viscera, such as an electrocautery needle, harmonic scalpel, and so on, can minimize this risk. A bipolar energy source may be chosen to avoid visceral and vascular injuries, but it is important not to forget that thermal injury can still occur with bipolar instruments. In recent years, the “active electrode monitoring” system has been very popular in laparoscopy for the detection of a current leak. This equipment will immediately turn off the system when an insulation leak is detected [28]. The usage of metal ports (by never using plastic collars on metal ports) is advised [12].

The conservative management of superficial bowel injuries with observation in a hospital, hyperalimentation and intravenous antibiotics has been investigated. Thompson and Wheeless reported that 6% of the cases with superficial electrocautery bowel injuries required open exploration due to acute perforation during the observation period. They concluded that intraoperative repair of the damaged bowel is significantly safer and should be performed in every suspicious electrocautery bowel injury [29].

Thermal damage of the bowels frequently results in more extensive damage than expected. A wide excision should be performed for the removal of all affected tissue. The injured site should be drained adequately, and the patient should be prescribed a proper antibiotic regimen [12].

Bipolar energy sources, the use of shielded instruments and the visualization of the hot part of the instrument can decrease electrocautery injuries during laparoscopy. The operating team and staff surgeon should check the insulation of monopolar or bipolar instruments before the procedure. A full investigation of the abdomen and operating field should be performed at the beginning and at the end of the procedure to rule out any visceral or vascular injuries [12]. The localization of all laparoscopic instruments inside the body should be checked by the operating team because the surgeon may not view the entire field. Ultrasonic devices can also protect patients against electrocautery-induced injuries because they do not apply electrical current inside the body. Baldie et al reported the robotic management of benign mid and distal ureteral strictures and compared the outcomes with a laparoscopic approach [30]. Sixteen patients underwent robotic mid and distal ureteral repair between 2008 and 2011 in their department, of which 13 were ureteral reimplantations and three were ureteroureterostomies. A symptomatic bowel injury was noted in one patient and repaired intraoperatively.

### Port site hernia (PSH)

Tanouchi et al were the first to report a PSH [31]. The incidence of PSH after laparoscopic procedures ranges between 0.14 and 22%. The most important factors related to the formation of PSH were older age, preexisting hernia, trocar design, trocar diameter, longer operation time and high BMI. The umbilicus was the most common port associated with incisional hernia [32]. PSH can lead to serious complications such as bowel obstruction, perforation and strangulation. If bowel function is delayed during the postoperative period, bowel perforation, ileus and PSH causing a bowel obstruction should be suspected. The diagnosis can be made with a CT scan, which will show a loop of bowel protruding through the abdominal fascia. Laparoscopic exploration or open surgery should be performed [12].

The use of trocar sizes less than 5 mm can prevent PSH. The sites of these ports are not closed in adults, but they should be closed in children. When removing the trocars, the site should be investigated for herniated structures or omentum. The diameter of the fascial defect caused by traditional bladed trocars is equal to the diameter of the trocar. On the other hand, this defect is only half of the diameter of the trocar, when non-bladed dilating trocars are used. Even 12 mm non-bladed dilating trocars may not require fascial closure because the diameter of the fascial defect will only be 6 mm in size [12].

Park et al reported a bowel herniation from the site of a 12 mm bladeless trocar in a 67-year-old woman with rectal cancer who underwent robotic colorectal surgery [33]. On seventh postoperative day, the hernia was diagnosed by an abdominal CT and was repaired laparoscopically.

### Bowel injuries by mechanical effects

Various laparoscopic instruments as well as robotic arms with no tactile feedback can cause severe mechanical injuries of the bowel. The injury type can be sharp or blunt. Mechanical injuries mostly occur outside the laparoscopic visual field due to the blind operation of the instruments or the blind dissection of the non-target tissues outside the surgery field. Similar to other bowel injuries, the management can be performed by laparoscopic intracorporeal suturing (even though the patient has not been prepared during the preoperative period). Bowel resection and a diverting colostomy are rarely required after this type of injury. If there is an extensive injury and bowel resection is necessary, a general surgeon should be invited to the operating room to decide the proper management strategy [12].

All tissue handling and instrument insertion into the peritoneal cavity should be performed with direct visual guidance [12]. The laparoscopic instruments should not be left inside the peritoneum when they are not being used. Especially during robot-assisted surgery, the fourth arm, when used, should always be placed in a secure, visible location, and clutching of the arms should be prevented. These precautions can prevent mechanical injuries.

### Other organ injuries

Injury to the stomach can occur specifically during a left side nephrectomy and adrenalectomy. If the perforation is small enough, it can be closed by intracorporeal laparoscopic suturing. The stomach should be decompressed with a nasogastric tube, and an abdominal drain should be placed adjacent to the repaired site [12].

Injury to the duodenum is a very serious complication due to the high morbidity associated with duodenal leakage. This injury occurs most during renal surgery of the right side and laparoscopic retroperitoneal lymphadenectomy. Intraoperative general surgery consultation is mandatory. Open conversion and repair of the injury site should be performed with the placement of an abdominal drain. It may sometimes necessitate resection of a segment of the duodenum and duodenojejunal anastomosis [12]. Small bowel injury during laparoendoscopic single site surgery for simple nephrectomy was reported in a 71-year-old man [34]. The injury was diagnosed by physical findings and CT during the postoperative period. The patient underwent an emergency exploratory laparotomy and a < 5 mm full thickness perforation of the duodenum and an accompanying 1 cm serosal injury were observed. The postoperative course was normal except for a right intraabdominal seroma which disappeared without any intervention.

Initial trocar insertion, Veress needle passage and non-monitored instruments that are outside of the surgeon’s field of vision can cause hepatic or splenic injuries. Organomegalies detected on CT before the procedure should prompt the operating team to perform lower abdominal or umbilical primary trocar placement [12].

Compression alone can be enough to manage minor injuries of the liver and spleen. Open surgical repair of the liver or splenectomy may be necessary for the uncontrolled bleeding of these organs [12]. In a multicenter study dealing with the operative safety and oncological outcome of laparoscopic radical nephrectomy for renal cell carcinoma > 7 cm, the data of 222 patients from five centers were investigated. All of the procedures were performed with a transperitoneal approach. They reported splenic injury in one patient, which was managed conservatively [35]. The presence of Riedel’s lobe of the liver presents two technical aspects: hilar exposure and intraperitoneal access [36]. For successful access, an initial supraumbilical approach or an open access should be used to avoid liver injury. Renal and hilar exposures were achieved after taking down the right lateral liver attachments which facilitates the medial retraction of the hepatic lobe.

Laparoscopic cholecystectomy after general surgery consultation is appropriate to correct gall bladder perforation, which is generally managed by cholecystectomy. Canes et al presented two patients out of 2,866 transperitoneal laparoscopic urologic procedures between 1997 and 2007, who sustained iatrogenic common bile duct injury [37]. One occurred during laparoscopic anterior pelvic exenteration and Indiana pouch diversion, and the other during laparoscopic partial nephrectomy.

Laparoscopic left side renal or adrenal surgery may cause pancreatic injury. The plane between the tail of the pancreas and Gerota’s fascia should be dissected very carefully to prevent trauma to the pancreas. If a pancreatic injury is suspected, an intraabdominal suction drain should remain in the left renal bed and fluid amylase levels should be checked to verify the pancreatic injury during the postoperative period. Conservative treatment with a drain will suffice for most superficial pancreatic injuries. However, a major pancreatic duct injury may necessitate distal pancreatectomy by laparoscopic surgery or open technique [12]. Winaikosol et al analyzed the perioperative data of 78 patients who underwent laparoscopic simple nephrectomy at their institution [38]. A transperitoneal and retroperitoneal approach was preferred for 38 and 40 cases, respectively. Prolonged ileus was observed in eight cases and pancreatic injury was noted in one patient who had a left side transperitoneal nephrectomy. The patient was managed conservatively with a drain.

Hawasli et al reported the outcomes of laparoscopic live donor nephrectomy in their institution after 6 years of performing this surgery. A minor mesenteric injury was observed in seven (4.2%) cases. All of these cases were recognized intraoperatively and were repaired laparoscopically. They reported no ureteral or bowel injuries [39].

### Effect of previous open abdominal surgery on complications

Previous abdominal surgery is a risk factor for bowel injuries during laparoscopic surgery. Parsons et al reported the effect of previous abdominal surgery with open or laparoscopic surgeries on secondary kidney laparoscopic procedures [40]. Of the 700 patients, 366 (52%) had never undergone abdominal surgery, 229 (33%) had undergone abdominal surgery in a different region and 105 (15%) had a history of abdominal surgery at the same anatomic location. There were no significant differences between the three groups in terms of complications and conversion rates.

### Influence of gastrointestinal complications on readmission

Many groups experienced in laparoscopic and robotic surgeries reported their readmission rates after partial nephrectomies [41-44]. In a recent article from the USA, the authors retrospectively reviewed the medical charts of 627 patients who underwent RPN at their institution. Twenty-eight (4.46%) patients were readmitted within 30 days of surgery. Postoperative bleeding was the most common complication causing readmission [41]. Khalifeh et al compared the outcomes of robotic and laparoscopic partial nephrectomy in 500 cases. The robotic group had significantly fewer intraoperative and postoperative complications (P < 0.001) when compared to the laparoscopic group [42]. Early discharge and readmission after robotic or laparoscopic partial nephrectomy were investigated in a recent article. The authors investigated the medical charts of 263 consecutive minimally invasive partial nephrectomies from 2003 to 2010. The primary endpoint of the study was successful implementation of the clinical pathway with discharge on postoperative day 1. They reported a 60% rate of successful discharge on postoperative day 1 and a readmission rate of 5% (12/263) [43]. A similar issue was reported in another study. The group investigated whether a single overnight stay was possible for patients undergoing RPN. The medical files of 150 consecutive patients who underwent 160 RPNs were investigated. They revealed a discharge rate of 97% on the first postoperative day, with a 2.7% rate of readmission within 30 days [44]. None of the aforementioned articles blamed gastrointestinal complications for readmissions to the hospitals.

### Rectal injury

Rectal injury is a rare, but serious complication that occurs at a rate of 1.7% during laparoscopic or robot-assisted radical prostatectomies [41, 42]. Rectal injury frequently occurs during the dissection of the base or apex of the prostate when the Denonvilliers’ fascia is not properly incised [12]. The clean, uncontaminated procedure becomes a contaminated procedure when a rectal injury occurs. Rectal injury increases the risk of septic complications such as wound infection, rectourethral fistula, peritonitis and death. In a large study by Guillonneau et al, 13 rectal injuries (1.3%) occurred during 1,000 transperitoneal laparoscopic radical prostatectomies [45]. The patients had no preoperative radiotherapy, hormone therapy or previous prostatic surgery. Eleven rectal injuries were intraoperatively diagnosed and primarily repaired. Nine of the 11 patients healed without colostomy. The other two patients were explored during the postoperative period because of fever and abdominal pain. One patient required the re-suturing of a small rectal defect, and the other required a colostomy. Rectal injury was diagnosed in two patients during the postoperative period. These patients had fever, abdominal distension and umbilical pain after 3 - 4 days. One of these patients had a small rectal perforation that was managed with a colostomy. The other patient was also managed with a colostomy but developed a rectourethral fistula during the third postoperative month. Perineal repair was performed for the rectourethral fistula.

Bowel complication during robotic-assisted laparoscopic radical prostatectomy (RALP) was reported in a recent article [46]. The group observed bowel injuries in only three (1.04%) out of 288 RALPs in their department between 2005 and 2011. The clinical stages of the cases were T1b, T1c and T2c. Two of the intraoperative injuries were diagnosed and repaired intraoperatively. One healed primarily without colostomy, but a rectourinary fistula developed in the other. The other case, which was diagnosed in the postoperative period due to atypical acute abdomen, required laparotomy and colostomy. No perioperative mortality was reported in these three cases.

The management of rectal injuries during laparoscopic procedures depends on the surgeon’s experience. Small perforations may be closed with laparoscopic reconstructive techniques, whereas larger rectal injuries may require colostomy and laparotomy.

If the rectal injury is diagnosed intraoperatively, the surgery field should be irrigated with saline or povidone iodine after removing the prostate tissue during radical prostatectomy. After prostatectomy, the margins of the rectal defect should be identified by digital rectal examination or a metallic bougie. The rectal wall and muscular layers of the defect should be clearly observed. The rectal wall is closed in two layers (inner mucosa and outer seromuscular layer) with continuous 3-0 polyglactinsutures. The rectal repair can be performed laparoscopically (traditional or robot-assisted), depending on the experience of the surgeon. The integrity of the repair should be checked by filling the rectum via a rectal catheter, generally placed immediately before surgery in selected cases. The pelvic cavity can be filled with sterile saline, and after filling the rectum with air via a catheter, bubbles in the field can be identified where there is leakage at the repair site. If no leakage is observed, vesicourethral anastomosis can be established. At the end of the procedure, generally two drains are placed: one posterior to the bladder near the injury site and another in the space of the Retzius. Broad spectrum antibiotics should be administered for 5 - 7 days. Oral liquids can be given the day after surgery, and a diet can be initiated after passing flatus [12].

### Hand-assisted laparoscopy

Hand-assisted laparoscopy requires a standard 6- to 8-cm incision at the beginning of the procedure, through which a hand-assisted device is inserted into peritoneum. This technique is generally preferred for donor nephrectomy. Pneumoperitoneum is established with the hand-assisted device. Access-related bowel and vascular complications are expected to be rare. However, herniation and wound infection are more common after this procedure [47]. Special attention should be taken to avoid protrusion of the bowel segments while closing the site.

Hand-assisted laparoscopy has low morbidity, but different types of complications can occur, including superior mesenteric artery syndrome [47, 48]. Kanemitsu et al reported a case of superior mesenteric artery syndrome after hand-assisted, laparoscopic left nephrectomy [48]. The patient was a 65-year-old man with left side renal cell carcinoma. No early complications were noted during the postoperative period, and he was discharged on the eighth postoperative day. He was admitted to the hospital 2 days after discharge for vomiting and abdominal pain. A CT scan revealed a narrow space between the superior mesenteric artery and aorta. The patient was diagnosed with superior mesenteric artery syndrome, and he was managed conservatively with total parenteral nutrition and a nasogastric tube. He was discharged on the 34th day after treatment.

## Conclusions

Gastrointestinal complications are rare and similar following laparoscopic and robotic urologic surgery. They can occur at anytime, from access to closure. Despite their infrequency, they can result in mortality. Appropriate surgical technique, surgeon experience and a high degree of vigilance are necessary throughout the robotic and laparoscopic surgery. The early recognition and management of gastrointestinal complications is very important. Unrecognized or delayed identification of gastrointestinal complications may cause sepsis and death. In our opinion, if there is a suspicion of gastrointestinal injury, general surgery consultation should be sought and a general surgeon should be invited to the operating room to discuss the correct management strategy.

## Abbreviations

FDA: Food and Drug Administration
CO_2_: carbon dioxide
mm: millimeter
BMI: body mass index
RPN: robotic partial nephrectomy
LESS-RN: laparo-endoscopic single site radical nephrectomy
CT: computerized tomography
PSH: port site hernia
RALP: robotic-assisted laparoscopic radical prostatectomy
